# Associations between parent–child relationship, and children’s externalizing and internalizing symptoms, and lifestyle behaviors in China during the COVID-19 epidemic

**DOI:** 10.1038/s41598-021-02672-7

**Published:** 2021-12-03

**Authors:** Fanxing Du, Li He, Mark R. Francis, Mark Forshaw, Kerry Woolfall, Qian Lv, Lu Shi, Zhiyuan Hou

**Affiliations:** 1 School of Public Health, NHC Key Laboratory of Health Technology Assessment, Fudan University, 130 Dong’an Road, Shanghai, 200032 China; 2 College of Physical Education and Sport, Beijing Normal University, Beijing, China; 3 Health Sciences Unit, Faculty of Social Sciences, Tampere University, Tampere, Finland; 4 School of Psychology, Faculty of Health, Liverpool John Moores University, Liverpool, UK; 5 Department of Public Health, Policy & Systems, University of Liverpool, Liverpool, UK; 6 Department of Public Health Sciences, College of Behavioral, Social and Health Sciences, Clemson University, Clemson, SC USA

**Keywords:** Psychology, Risk factors

## Abstract

To investigate associations between parent–child relationships, children’s externalizing and internalizing symptoms, and lifestyle responses to the COVID-19 epidemic, we conducted an online survey of a random, representative sample of residents with children aged 3–17 years during mid-March 2020 in Wuhan and Shanghai, China. A total of 1655 parents and children were surveyed with a response rate of 80.1% in the survey. During the epidemic, the frequency of children enquiring about the epidemic (AOR = 1.46, 95% CI: 1.04, 2.06), parents explaining the epidemic to them (AOR = 2.87, 95% CI: 1.80, 4.58), parents expressing negative emotions in front of them (AOR = 2.62; 95% CI = 2.08–3.30), and parents with more irritable attitudes (AOR = 1.93; 95% CI = 1.33–2.81) were significantly associated with children’s externalizing symptoms. For internalizing symptoms, significant associations were found with worse parent–child closeness (AOR = 2.93; 95% CI = 1.80–4.79), the frequency of parents expressing negative emotions in front of them (AOR = 2.64; 95% CI = 1.68, 4.12), and more irritable attitudes (AOR = 2.24; 95% CI = 1.42–3.55). We also found that each indicator of parent–child relationships had the significantly similar associations with children’s lifestyle behaviors. These findings suggest that improving parents’ attitudes towards their children and parent–child closeness during the epidemic, especially among parents with lower educational levels, are important to ensure the wellbeing of children.

## Introduction

In late 2019, a novel coronavirus disease (COVID-19) emerged in Wuhan city and spread rapidly across China^1^. To control the transmission of COVID-19, the Chinese government implemented strict quarantine measures and issued the highest-level public health alert in the country. As a result, in Wuhan city, the epicenter of the COVID-19 epidemic, a complete lockdown was adopted from January 23^2^ to April 8, 2020^3^, whereas in Shanghai, a megacity significantly affected by imported COVID-19 cases from Wuhan, a partial lockdown was adopted, and it activated the highest-level public health emergency response on January 24, 2020^4^. This lockdown in Shanghai was lifted on March 24, 2020^5^.

The severity of the COVID-19 epidemic and consequent quarantine measures, including stay-at-home-orders, school closure, delayed return to workplaces, and suspension of mass gathering, may lead to important psychological problems and lifestyle changes among adults as well as children^6^. During the epidemic, in Wuhan and Huangshi cities, 18.9% and 22.6% of primary school students reported symptoms of anxiety and depression, respectively, and the prevalence of behavioral problems among their peers varied from 4.7 to 10.3% during home quarantine compared to normal times^7,8^. Moreover, among Chinese children aged 7–18 years old, the prevalence of spending longer than 3 h per day on the internet increased from 25.8% during normal times to 58.2% during the epidemic^9^. Evidence from a longitudinal analysis involving 14 countries^10^ showed that, during the COVID-19 pandemic, children spent almost one hour more on sedentary screen time per day, went to bed 34 min later, woke up 60 min later and spent less time outdoors compared to non-pandemic times. These findings point to the possibility that the COVID-19 pandemic and consequent series of quarantine measures may exacerbate emotional or behavioral disorders and unhealthy lifestyles, which should be treated as early as possible to avoid long-term ill effects on the healthy development of children.

According to the family systems theory, parents are known to play a vital role in developing and expressing children’s emotions and behaviors^11^. With the implementation of stay-at-home-orders, school closure, and delays in parents’ return to workplaces during the epidemic, children’s social interactions are generally limited to their closest family members^6^, and the influence of parents on children’s emotional and behavioral responses to the epidemic needs attention. During the epidemic, Chinese adults had longer periods of inactivity, increased screen time, and psychological health problems than before^12,13^. In addition, in China and globally, children of parents who were more stressed were less likely to meet the recommended guidelines of physical activity, sedentary behavior, and sleep than those whose parents were less stressed during the epidemic^8,10^. Also, all negative emotions (e.g., fear, anger, loneliness, and sadness) of parents were positively associated with controlling parenting behaviors (e.g., wanting to control whatever the child does)^14^. However, there is limited evidence on the influence of other parenting factors such as their communication frequency, attitudes towards children, and the closeness between parents and children on children’s emotional, behavioral problems, and lifestyle behaviors during the epidemic.

During the epidemic, interactions between parents and children, and their closeness and attitudes towards each other, may change due to the different stressors parents face, which can influence children’s emotions and behaviors. Therefore, it is important to quantify the influence of parent–child relationships on children’s emotions and behaviors during the epidemic to design tailored interventions. Our study aimed to 1) investigate associations between parent–child relationships (including communication, attitude, and closeness) and children’s internalizing, externalizing symptoms, and lifestyle changes during the epidemic; and 2) explore potential inequities in variables above-mentioned by socio-economics levels. Thus, this study would fill the gap in this research area and help develop interventions to better promote the wellbeing of children during health emergencies in the Chinese context.

## Methods

### Study design

**Figure 1:**
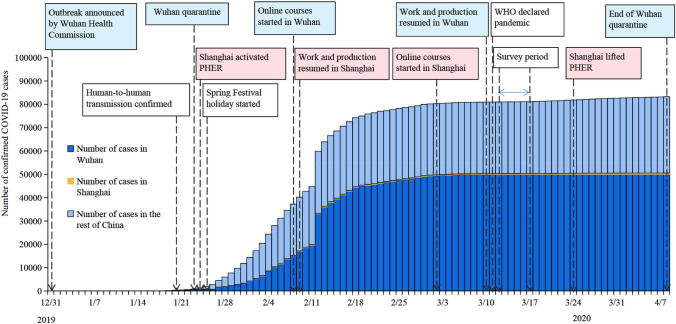
The timeline of the COVID-19 epidemic progression and quarantine measures in Wuhan and Shanghai. Note: PHER: public health emergency response.

An online survey of randomly recruited residents with children aged 3–17 years in Wuhan and Shanghai was conducted during March 12–17, 2020. These two cities were selected to represent different levels of challenges posed by the COVID-19 epidemic to parents and children. Figure 1 illustrates the timeline of the epidemic progression and quarantine measures deployed. The COVID-19 cases reached around 5000 in Wuhan and 350 in Shanghai during our survey period. Wuhan was put in quarantine on January 23 and ended its quarantine on April 8; Shanghai activated the highest-level public health emergency response (PHER) on January 24, which was lifted on March 24, 2020. Both Wuhan and Shanghai isolated suspected patients, closed schools and entertainment venues, and suspended public gatherings. At the same time, Shanghai did not suspend intra-city public transport (bus and subway) and prohibit inter-city travel compared to the lockdown measures in Wuhan^15^. The Spring Festival national holiday started on January 24, and work and production resumed for parents on February 9 and March 10, 2020 in Shanghai and Wuhan, respectively. Schools reopened for children from early May 2020, and online courses were taken during the school closure.

Our target population was children aged 3–17 years, who, along with their parents, resided in Wuhan or Shanghai for at least six months prior to the survey. The sample size (n = 800 for each city) was calculated to provide a sampling error of 3%, and proportional quota sampling was done to ensure that respondents were a population-based sample, representative of children’s age and gender distributions in the cities^16,17^. Respondents were sampled using the proportions of children in different age groups and by gender using estimates from a recent census^18^, and survey sampling continued until the proportions of children in the age and gender quotas were achieved in Wuhan and Shanghai. Based on a national database with emails and household information registered, residents with children were randomly selected to receive the survey link, and informed consent was obtained at the beginning of the survey. Parents were asked to complete the questionnaire, and if they had a child aged ten years or older, the child was asked to self-report their emotional responses and lifestyle behavior before and during the COVID-19 epidemic. In total, 2960 parents accepted the invitation to participate in the survey, and 2065 parents with children aged 3–17 years were eligible. Among them, 410 either did not complete the questionnaire or had missing data that disqualified their inclusion. Thus, our final analytical sample consisted of 816 respondents in Wuhan and 839 respondents in Shanghai, and the effective response rate for the survey was 80.1% (1,655/2,065).

### Measures

#### Parent–child relationships during the COVID-19 epidemic

Parent–child relationships during the COVID-19 epidemic were assessed by (1) the frequency of parent–child communications about the COVID-19 epidemic, which is measured by the following three parent-reported frequencies of children actively asking their parents about the epidemic, parents explaining about the epidemic to their children, and parents expressing negative sentiments or discussing the epidemic in the presence of their children; (2) the change of parents’ attitude towards their child during the epidemic compared to the usual; and (3) changes in parent–child closeness during the epidemic compared to normal times. The frequencies of parent–child communications about the COVID-19 epidemic were assessed on a 5-point Likert scale with response options ranging from “never” to “always”. We dichotomized these variables due to the low number of responses for specific response options for each variable. There were low numbers of “Never” responses for the “Frequency of children actively asking about the epidemic” and “Frequency of parent explaining the epidemic to children” variables, and low numbers of “Always” responses to the “Frequency of parent expressing negative sentiments or discussed the epidemic in their child’s presence” variable. For the analysis, options of “always,” “usually,” and “sometimes,” were categorized as “yes”, and response options of “rare” and “never” were categorized as “no”. The “Yes” category for these options meant that children actively asked about the epidemic, parents explained about the epidemic to their children, and parents expressed negative sentiments or discussed the epidemic in their child’s presence; the “no” category means that they never or rarely initiated these communications.

#### Children’s internalizing symptoms during the COVID-19 epidemic

Children’s internalizing symptoms (i.e., emotional problems) during the COVID-19 epidemic were measured using the Emotional Symptoms Scale (ESS), which was part of the Strengths and Difficulties Questionnaire (SDQ)^19^. The ESS is a brief scale including a parent version and a child/adolescent version with demonstrated reliability and validity for use among children/adolescents aged 3–17 years^20^. For children aged 3–10 years, parents were asked to proxy-report the emotional scale for children in the present study, while for children/adolescents aged 11–17 years, children were asked to self-report responses to the questions on the emotional scale. The ESS comprised five items, each of which is scored on a three-point scale. For example, “During COVID-19 outbreak (before the start of school), has the child/adolescent had many worries or often seemed worried?”. The responses ranged from 0 to 2 for each item in the ESS (0 = not true, 1 = somewhat true, 2 = certainly true). In total, the score for the ESS scale ranged from 0–10 for all five items. Based on a previous study, a summary score of 5–10 in the parent version and 7–10 in the child/adolescent version indicated a substantial risk of clinically significant emotional problems in children^19^.

#### Children’s externalizing symptoms during the COVID-19 epidemic

Additionally, according to recommendations by the American Psychological Association, the frequencies of aggressive or stubborn behavior (1 item) and tantrums or meltdowns behaviors (1 item) were used to measure the externalizing symptoms (i.e., behavioral problems) children experienced during the epidemic^21^. Children who reported “never” to both the questions were categorized as not having behavioral problems, and all the other responses were categorized as having behavioral problems^21^.

#### Children’ lifestyle behaviors before and during the COVID-19 epidemic

Twelve questions were used to measure children’s lifestyle behaviors before (i.e., previous winter break) and during the COVID-19 epidemic in the survey, including daily screen time (hours per day, 1 item), days per week and daily time of moderate to vigorous physical activity (days per week and minutes per day, 2 items), sleep hours per night (1 item), and the sleep quality (2 items) before and during the epidemic. The questions about sleep quality were based on the Sleep Disturbance Scale for Children (i.e. difficulty getting to sleep and frequency of waking up at night)^22^. For example, “During the COVID-19 outbreak (before the start of school), did the child/adolescent have difficulty getting to sleep at night?” Response options included never, 1–2 times a month or less, 1–2 times a week, 3–5 times a week, and nearly every day. One more question was used to assess the frequency of going outside per week during the epidemic.

**Figure 2:**
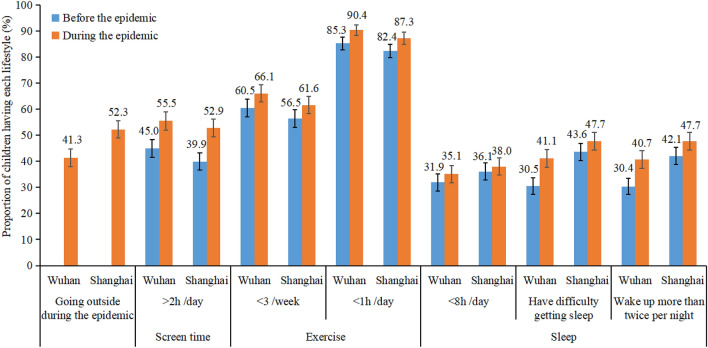
Comparing the proportions of children having unhealthy lifestyles before and during the COVID-19 epidemic. Note: Percentage (%) and 95% confidence interval were presented.

According to the World Health Organization recommendations on physical activity, sedentary behavior, and sleep for good health^23,24^, children who (1) had a screen time of more than 2 h per day, (2) exercised less than three times per week, (3) did moderate to vigorous physical activity less than 1 h per day, (4) slept less than 8 h per night, (5) had difficulty getting to sleep and (6) woke up more than twice per night were categorized as having “unhealthy” lifestyle behaviors for the analyses (Fig. 2). To measure changes in children’s lifestyles, we compared the proportion of children who had unhealthy lifestyle behaviors during the previous winter break and the COVID-19 epidemic.

In addition, children aged 11–17 years were encouraged to self-report their lifestyle, while parents were asked to proxy-report for their children under 11 years. Details of the survey questionnaire are available in Appendix A.

#### Socio-demographic characteristics

The characteristics of children included gender, age, and whether they experienced fever symptoms during the epidemic. The characteristics of families included household size, educational level of parents, family economic status (income of parents), parents’ employment status, and whether there were confirmed COVID-19 cases in the neighborhood.

### Statistical Analysis

Chi-square tests or Fisher’s exact tests (if expected frequency < 5) were used to compare differences in the parent–child relationships, and children’s internalizing symptoms, externalizing symptoms, as well as lifestyle changes during the epidemic between Wuhan and Shanghai (Tables 1, 2, and 3).

We also investigated potential inequities in these variables abovementioned by parents’ socio-economic status. For comparison, ratios were generated by dividing children’s outcome proportions for the lowest and highest socio-economic groups. A ratio of 1 indicates no inequity between the groups (Table 3 and Appendix B Table B1). The lowest and highest socio-economic groups were without a bachelor degree vs. bachelor degree or above, low vs. high income levels, and unemployed vs. office-working employments. p-values were calculated using Chi-square tests or Fisher’s exact tests (if expected frequency < 5). In the equity analysis, children’s lifestyle changes were measured as the proportions of children who had more screen time, less exercise, less sleep time, and more often had difficulty getting sleep or woke up more than twice per night during the epidemic than normal times.

Multivariate logistic regressions were used to estimate associations between the parent–child relationship indicators and children’s internalizing or externalizing symptoms and their lifestyle changes during the epidemic. We used six dichotomous variables to measure the children’s emotional responses and lifestyle changes during the epidemic. If children/adolescents had abnormal emotions or stress symptoms during the COVID-19 epidemic, they were coded as “yes” (with the others coded as “no”) for the two variables, respectively: “abnormal emotions” and “stress symptoms”. If they had more screen time, less exercise, less sleep time or worse sleep quality during the epidemic than during last winter break, they were coded as “yes” (with the others coded as “no”) for the other four variables, respectively: “more screen time”, “less exercise”, “less sleep” and “worse sleep quality”. A total of six logistic regressions were performed on the six dependent variables above, respectively. Each regression was controlled for child (gender, age, fever symptoms or going outside during the epidemic) and family characteristics (education, family economic status, employment status, having COVID-19 cases in neighborhood), and respondent type (father, mother or child/adolescent), and the location of residence (Table 4). Proportions, adjusted odds ratios (AOR) and 95% confidence intervals (CI) are reported. All statistical analyses were performed using Stata 14.0 (Stata Corp LP, College Station, TX).

### Ethical approval

The study was approved by the institutional review board at School of Public Health, Fudan University (IRB#2020-01-0801-S). We received written informed consent from at least one of parent or guardian for all children participating in the survey. All methods were performed in accordance with the relevant guidelines and regulations proposed by the institutional review board at School of Public Health, Fudan University.

## Results

### Participants’ characteristics

Table 1 shows that the characteristics of children in Wuhan and Shanghai were not significantly different in terms of gender, age distribution, and whether they experienced fever or cough symptoms during the epidemic (p > 0.05). The mean age of children in Wuhan was 10.5 years (Standard Deviation = 4.67), which was similar to Shanghai (10.6 years, SD = 4.68) (P = 0.596). Regarding the family characteristics, there were no significant differences in the family household size and educational level of parents between Wuhan and Shanghai. However, significant differences between the two cities were observed by the families’ economic status, employment status of parents, and the occurrence of COVID-19 cases in the neighborhood (p < 0.001). 1244 (75.2%) parents and 411 (24.8%) children responded to questions about children’s internalizing and externalizing symptoms, and lifestyle behaviors, respectively. Among the 1244 children whose parents completed the questionnaire, 491 were aged 11–17 years.

**Table 1:** Socio-demographic characteristics of participants in Wuhan and Shanghai, n (%).

Characteristics	Total (n = 1655)	Wuhan (n = 816)	Shanghai (n = 839)	p value*
***Characteristics of children***				
**Gender of children**				0.751
Male	830 (50.2)	406 (49.8)	424 (50.5)	
Female	825 (49.9)	410 (50.3)	415 (49.5)	
**Age of children, years**				0.838
3–5	321 (19.4)	160 (19.6)	161 (19.2)	
6–9	432 (26.1)	217 (26.6)	215 (25.6)	
10–14	359 (21.7)	180 (22.1)	179 (21.3)	
15–17	543 (32.8)	259 (31.7)	284 (33.9)	
Age of children (years), mean (Standard Deviation)	10.5 (4.67)	10.5 (4.67)	10.6 (4.68)	0.596
**Children experienced fever symptoms during the epidemic**				0.677
Yes	118 (7.1)	56 (6.9)	62 (7.4)	
No	1537 (92.9)	760 (93.1)	777 (92.6)	
***Family characteristics***				
**Household size**, mean (Standard Deviation)	3.5 (1.30)	3.5 (1.26)	3.5 (1.34)	0.394
**Education level of parent**				0.738
Three years college or below	430 (26.0)	215 (26.4)	215 (25.6)	
Bachelor or above	1225 (74.0)	601 (73.7)	624 (74.4)	
**Family economic status**				< 0.001
Low	188 (11.4)	79 (9.7)	109 (13.0)	
Middle	886 (53.5)	410 (50.3)	476 (56.7)	
High	581 (35.1)	327 (40.1)	254 (30.3)	
**Employment status**				< 0.001
Work at office	644 (38.9)	273 (33.5)	371 (44.2)	
Work at home	637 (38.5)	311 (38.1)	326 (38.9)	
Have not returned to work or unemployed	374 (22.6)	232 (28.4)	142 (16.9)	
**COVID-19 cases in neighborhood**				< 0.001
Yes	365 (22.0)	277 (33.9)	88 (10.5)	
No or unclear	1290 (78.0)	539 (66.1)	751 (89.5)	
***Respondent***				
**Respondent for overall questionnaire**				0.099
Mother	1077 (65.1)	547 (67.0)	530 (63.2)	
Father	578 (34.9)	269 (33.0)	309 (36.8)	
**Respondent for questions about children’s emotional responses and lifestyle**				0.226
Parent	1244 (75.2)	624 (76.5)	620 (73.9)	
Children	411 (24.8)	192 (23.5)	219 (26.1)	

*p value are from Chi square or fisher’s exact tests (when applicable).

### Parent–child relationships, children’s internalizing and externalizing symptoms, and lifestyle changes during the COVID-19 epidemic

During the COVID-19 epidemic, 76.7% (626/816) in Wuhan and 84.7% (711/839) of children in Shanghai always, usually, or sometimes asked about the epidemic actively (Table 2 Panel A). A majority of parents always, usually, or sometimes explained the epidemic to their children in Wuhan (83.8%, 684/816) and Shanghai (93.0%, 780/839). Nearly half of the parents (42.5%, 347/816 in Wuhan and 49.2%, 413/839 in Shanghai) always, usually, or sometimes expressed negative emotions or discussed the epidemic in the presence of their children. 51% and 56.6% of parents reported being more patient towards their children than usual in Wuhan and Shanghai, respectively. 53.3% and 60.9% of parents in Wuhan and Shanghai reported having better parent–child closeness during the epidemic than usual.

A small proportion of children (8.0%, 65/816 in Wuhan and 11.7%, 98/839 in Shanghai) had a substantial risk of clinically significant emotional problems (Table 2 Panel B). And 39.5% of children in Wuhan and 58.4% in Shanghai had behavioral problems such as aggressive or stubborn behavior and tantrums or meltdowns during the epidemic (p < 0.001).

**Table 2:** Parent–child relationship, and children’s internalizing and externalizing symptoms during the COVID-19 epidemic in Wuhan and Shanghai, n (%).

	Total sample (n = 1655)	Wuhan (n = 816)	Shanghai (n = 839)	p value*
**Panel A: Parent–child relationship indicators**				
**Frequency of children actively asking about the epidemic**				< 0.001
Always	303 (18.3)	145 (17.8)	158 (18.8)	
Usual	565 (34.1)	269 (33.0)	296 (35.3)	
Sometimes	469 (28.3)	212 (26.0)	257 (30.6)	
Rare	222 (13.4)	142 (17.4)	80 (9.5)	
Never	96 (5.8)	48 (5.9)	48 (5.7)	
**Frequency of parent explaining the epidemic to children**				< 0.001
Always	566 (34.2)	262 (32.1)	304 (36.2)	
Usual	580 (35.1)	274 (33.6)	306 (36.5)	
Sometimes	318 (19.2)	148 (18.1)	170 (20.3)	
Rare	183 (11.1)	129 (15.8)	54 (6.4)	
Never	8 (0.5)	3 (0.4)	5 (0.6)	
**Frequency of parent expressing negative sentiments or discussed the epidemic in their child’s presence**				0.001
Always	130 (7.9)	73 (9.0)	57 (6.8)	
Usually	217 (13.1)	88 (10.8)	129 (15.4)	
Sometimes	413 (25.0)	186 (22.8)	227 (27.1)	
Rare	541 (32.7)	269 (33.0)	272 (32.4)	
Never	354 (21.4)	200 (24.5)	154 (18.4)	
**Parents’ attitude towards children**				0.013
More irritable	258 (15.6)	147 (18.0)	111 (13.2)	
Unchanged	506 (30.6)	253 (31.0)	253 (30.2)	
More patient	891 (53.8)	416 (51.0)	475 (56.6)	
**Parent–child closeness**				0.008
Worse	177 (10.7)	95 (11.6)	82 (9.8)	
Unchanged	532 (32.2)	286 (35.1)	246 (29.3)	
Better	946 (57.2)	435 (53.3)	511 (60.9)	
**Panel B: Children’s internalizing & externalizing symptoms**				
Having emotional problems^#^	163 (9.9)	65 (8.0)	98 (11.7)	0.011
Having behavioral problems	812 (49.1)	322 (39.5)	490 (58.4)	< 0.001

*p value are from Chi square or fisher’s exact tests (when applicable).

^#^One’s SDQ-Emotional Symptoms Score > 4 for parent version or > 6 for child version was considered to have substantial risk of clinically significant emotional problems (in short, having emotional problems).

Figure 2 presents the comparisons of children’s lifestyles before and during the COVID-19 epidemic. About 58.7% (n = 479) and 47.7% (n = 400) of the children in Wuhan and Shanghai never went outside during the COVID-19 epidemic. In Wuhan and Shanghai, the prevalence of children not meeting the recommendations for different lifestyle behaviors was 55.5% and 52.9% for screen time, 90.4% and 87.3% for daily moderate to vigorous physical activity, and 35.1% and 38.0% for sleep time during the epidemic, respectively

.

### Inequity in parent–child relationships, children’s internalizing and externalizing symptoms, and lifestyle changes during the COVID-19 epidemic

As shown in Table 3, there was a significant inequity between low-and high-level education groups in the proportion of children who actively asked about the epidemic (ratio = 1.10, p = 0.023), and parents who explained the epidemic to children (ratio = 1.11, p = 0.003) in Wuhan, but not in Shanghai. However, in both cities, more parents expressed negative sentiments or discussed the epidemic in the presence of their children when they were at a low level of education (Wuhan: ratio = 1.31, p = 0.002; Shanghai: ratio = 1.27, p = 0.001). Moreover, parents with lower educational attainment had a more irritable attitude towards their children and worse closeness during the epidemic in both cities (p ≤ 0.001). Children of parents with lower educational attainment were also more likely to have emotional problems in Wuhan (ratio = 1.86, p = 0.009) and Shanghai (ratio = 2.00, p < 0.001), but no significant differences by their parents’ educational levels were found for screen time, exercise and sleep quality of children (p > 0.05). The proportions of children going outside during the epidemic were significantly higher among parents in the low-level educational groups in Shanghai (ratio = 1.25, p = 0.002) and Wuhan (ratio = 1.28, p = 0.005). Similar results were observed for parents stratified by family economic status and employment status (Appendix B Table B1).

**Table 3:** Equity (ratio between the comparison groups) in parent–child relationships, children’s internalizing and externalizing symptoms, and lifestyle changes during the COVID-19 epidemic.

	Ratio between low- and high-education groups*			
	Wuhan	p value	Shanghai	p value
**Panel A: Parent–child relationship indicators**				
Children actively asked about the epidemic**	1.10	0.023	1.06	0.086
Parent explained the epidemic to children**	1.11	0.003	1.02	0.335
Parent expressed negative sentiments or discussed the epidemic in their child’s presence**	1.31	0.002	1.27	0.001
More irritable attitude towards children	2.60	< 0.001	1.83	0.001
Worse parent–child closeness	2.74	< 0.001	3.05	< 0.001
**Panel B: Children’s internalizing & externalizing symptoms**				
Having emotional problems^#^	1.86	0.009	2.00	< 0.001
Having externalizing symptoms	1.09	0.402	1.17	0.013
**Panel C: Children’s lifestyle changes during the epidemic compared with normally**				
Going outside during the epidemic	1.28	0.005	1.25	0.002
More screen time per day	1.18	0.131	0.94	0.545
Less exercise per week or per day	1.19	0.081	0.85	0.132
Less sleep at night	1.24	0.163	0.87	0.397
More often had difficulty getting sleep or woke up > twice per night	1.25	0.054	0.77	0.054

*Ratios generated by dividing the proportion of the group without bachelor degree by the group with bachelor or above. The ratios indicate the gap between the comparison group—where 1 implies no gap, and p value are from Chi square or fisher’s exact tests (when applicable).

**This is “Yes” category which includes response options of “always,” “usually,” and “sometimes” for three questions on the frequencies of parent–child communications about the COVID-19 epidemic.

^#^One’s SDQ-Emotional Symptoms Score ≥ 5 for parent version or ≥ 7 for child version was considered to have substantial risk of clinically significant emotional problems (in short, having emotional problems).

### Association between parent–child relationships and children’s internalizing and externalizing symptoms, as well as lifestyle changes during the COVID-19 epidemic

Table 4 showed that after controlling for child and family characteristics, children who enquired about the epidemic were more likely to have externalizing symptoms (AOR = 1.46, 95% CI: 1.04, 2.06) and less likely to have a lower duration of sleep (AOR = 0.64, 95% CI: 0.44, 0.94). Children whose parents explained the epidemic to them were more likely to have externalizing symptoms (AOR = 2.87, 95% CI: 1.80, 4.58), more screen time (AOR = 1.68, 95% CI: 1.12, 2.54), less sleep (AOR = 2.52, 95% CI:1.48, 4.30), and worse sleep quality (AOR = 2.45, 95% CI: 1.49–4.01). Compared to children whose parents rarely or never expressed negative sentiments or discussed the epidemic, children whose parents expressed negative emotions or discussed the epidemic in the presence of them had a significantly higher odds of emotional problems (AOR = 2.64; 95% CI = 1.68, 4.12), externalizing symptoms (AOR = 2.62; 95% CI = 2.08–3.30), and worse sleep quality (AOR = 1.34; 95% CI = 1.06–1.71) during the epidemic.

**Table 4:** Associations between the parent–child relationship indicators, children’s internalizing and externalizing symptoms, and lifestyle changes during the COVID-19 epidemic, multivariate logistic regression.

Parent–child relationship indicators	Internalizing symptoms	Externalizing symptoms	Lifestyle changes			
			More screen time	Less exercise	Less sleep	Worse sleep quality
**Children actively asked about the epidemic**	2.04	1.46*	0.93	1.00	0.64*	1.31
(Ref. rare or never)	(0.93–4.49)	(1.04–2.06)	(0.67–1.29)	(0.73–1.38)	(0.44–0.94)	(0.90–1.90)
**Parent explained the epidemic to children**	0.98	2.87**	1.68*	1.36	2.52**	2.45**
(Ref. rare or never)	(0.39–2.48)	(1.80–4.58)	(1.12–2.54)	(0.92–2.00)	(1.48–4.30)	(1.49–4.01)
**Parent expressed negative sentiments or discussed the epidemic**	2.64**	2.62**	1.09	1.11	1.14	1.34*
(Ref. rare or never)	(1.68–4.12)	(2.08–3.30)	(0.87–1.36)	(0.89–1.39)	(0.87–1.49)	(1.06–1.71)
**More irritable attitude towards children**	2.24**	1.93**	1.50*	1.01	1.72**	1.50*
(Ref. unchanged or more patient)	(1.42–3.55)	(1.33–2.81)	(1.08–2.09)	(0.72–1.40)	(1.17–2.51)	(1.07–2.10)
**Worse parent–child closeness**	2.93**	1.18	1.02	1.60*	0.77	2.36**
(Ref. unchanged or better)	(1.80–4.79)	(0.76–1.83)	(0.69–1.52)	(1.09–2.35)	(0.48–1.24)	(1.59–3.49)
**Age of children, years (Ref: 3–5)**						
6–9	1.42	0.73	1.08	0.98	0.52**	0.94
	(0.86–2.35)	(0.52–1.02)	(0.7–1.48)	(0.71–1.36)	(0.35–0.77)	(0.67–1.33)
10–14	0.87	0.69	0.94	1.20	0.58*	1.13
	(0.46–1.66)	(0.4–1.00)	(0.66–1.34)	(0.84–1.70)	(0.38–0.89)	(0.77–1.65)
15–17	0.77	0.43**	1.03	1.36	0.81	0.83
	(0.42–1.40)	(0.30–0.62)	(0.73–1.46)	(0.97–1.90)	(0.54–1.20)	(0.57–1.21)
**Respondent types (Ref. children)**						
Mother	3.12**	0.92	0.60**	0.67*	0.43**	0.68*
	(1.53–6.34)	(0.66–1.28)	(0.44–0.83)	(0.50–0.91)	(0.30–0.62)	(0.49–0.95)
Father	4.95**	1.35	0.72	0.60**	0.71	0.75
	(2.37–10.33)	(0.93–1.97)	(0.50–1.02)	(0.42–0.85)	(0.47–1.07)	(0.51–1.10)
**Observations**	1,655	1,655	1,655	1,655	1,655	1,655

Adjusted odds ratios with 95% confidence intervals were obtained from multivariate logistic regressions. We controlled for children characteristics (gender, age, fever symptoms or going outside during the epidemic), family characteristics (education, income, employment status, having COVID-19 cases in neighborhood), respondent types, and location. Significance level: **p < 0.01; *p < 0.05. Ref. means the reference group.

In addition, children with parents who became more irritable during the epidemic had a significantly higher odds of emotional problems (AOR = 2.24; 95% CI = 1.42–3.55), externalizing symptoms (AOR = 1.93; 95% CI = 1.33–2.81), more screen time (AOR = 1.50; 95% CI = 1.08–2.09), less sleep (AOR = 1.72; 95% CI = 1.17–2.51), and worse sleep quality (AOR = 1.50; 95% CI = 1.07–2.10) during the epidemic. Worse parent–child closeness during the epidemic was also positively associated with emotional problems (AOR = 2.93; 95% CI = 1.80–4.79), less physical activity (AOR = 1.60; 95% CI = 1.09–2.35), and worse sleep quality (AOR = 2.36; 95% CI = 1.59–3.49) during the epidemic.

## Discussion

Our study observed significant associations between different parent–child relationship indicators and children’s internalizing and externalizing symptoms, as well as lifestyle responses to the COVID-19 epidemic in China. More than 80% of parents or children explained or asked about the COVID-19 epidemic, 46% of parents expressed negative sentiments or discussed the epidemic in their child’s presence, and a little above 10% of parents had a more irritable attitude and reported worse parent–child closeness during the epidemic. Around 10% of children were at risk of clinically significant emotional problems, and about half of the children had externalizing symptoms during the epidemic. Our study also found a high prevalence (30% ~ 90%) of unhealthy lifestyle behaviors both at normal times (before the epidemic) and during the epidemic, with a higher prevalence during the epidemic. Moreover, inequities in parent–child relationship indicators, children’s emotional or behavioral problems, and time spent outside was observed among parents with differing educational levels during the epidemic.

Firstly, our survey found a high prevalence of inactive children even during normal times (before the COVID-19 epidemic), concurring with the findings of earlier studies. In a pooled analysis of 298 population-based surveys, the overall prevalence of insufficient physical activity was 84.3% among Chinese adolescents aged 11–17 years in 2016^25^, and in another study with a sample of 131,859 Chinese students aged 7–19 years, about 35% of children exceeded the daily recommended screen viewing time^26^. Moreover, we found that screen viewing time and physical activity levels worsened during the epidemic (compared to normal times) in Wuhan and Shanghai. This finding was consistent with previous research in other countries^10^ and highlights potential challenges to China achieving the WHO target of a 15% relative reduction in insufficient physical activity for children by 2030, due to the COVID-19 epidemic^27^. Furthermore, lesser hours of sleep and worse sleep quality were observed among children during the epidemic. The proportion of children sleeping less than 8 h during the epidemic increased by 2% and 3% in Shanghai and Wuhan, respectively. Children’s sleep, physical activity, and screen time are often found to be interacted, where more screen time and less time spent outdoors are adversely associated with sleep outcomes^28,29^. Therefore, a multi-tiered approach involving the society, school, and family is required to promote better mental and physical health for children and adolescents during the epidemic and similar public health emergencies^30^.

Secondly, the frequency of parent–child communication was found to be negatively associated with children’s mental or behavioral health, whereas parental patient attitudes or closeness with their children were positively associated with children’s mental or behavioral health. During the epidemic, if parents’ attitudes towards their children were unchanged or better than usual, children were significantly less likely to have unhealthy lifestyle behaviors except for the low moderate to vigorous physical activities. Meanwhile, if parents remained close to their children, children had more moderate-to-vigorous physical activity and decreased odds of emotional problems and worse sleep quality. These findings suggest that if parents are patient and remain close to their children, their children are more likely to have a healthier lifestyle during the epidemic. However, unlike the above findings, we found that a higher frequency of parents explaining the epidemic to their children was associated with longer screen time, less sleep, worse sleep quality, and more behavioral problems in children. Moreover, a higher frequency of parents expressing negative sentiments or discussing the epidemic in front of children may have detrimental effects on the children’s sleep quality, and internalizing or externalizing symptoms. Similarly, Liu et al. also found that children of parents with anxiety symptoms were associated with increased risks of emotional symptoms and hyperactivity-attention disorders, and peer problems^8^. These findings might imply that qualities like parental attitudes or closeness with their children and not the quantity of parent–child communication is more important for children’s mental or behavioral health.

The spread of misinformation and rumors about the epidemic and excessive exposure to negative information may increase parents’ and children’s fears and anxieties. The findings of the associations between the parent–child relationship indicators and outcomes concur with the view that parents who lack appropriate skill or guidance to help themselves and their children face greater adversity during a public health emergency. A previous study also found similar results for adults; adults who focused more on the epidemic were more likely to develop anxiety symptoms^31^. Family systems theory suggests that children and adolescents in families are strongly influenced by marital problems and poor parenting^11^. If parents have psychological distress, they are likely to transmit their distress during interactions with their children and create a negative atmosphere which could lead to persistent or delayed-onset distress symptoms^30^. Many studies have illustrated that mental health is worst among children whose caregivers have experienced adverse mental health outcomes from disasters^32–34^. For example, a recent study demonstrated that parents who experienced more fears related to the epidemic were more likely to have maladaptive parenting behaviors that put children at increased risk for adverse mental health outcomes^14^. Therefore, health education should be provided to parents to improve their knowledge about the epidemic and reinforce good parenting skills to improve parent–child relationships. Parents should be educated with scientific information to generate awareness about the importance of healthy lifestyles, such as ongoing physical activity and reduced screen time, especially during the COVID-19 epidemic. In addition, acknowledging the fear or stress parents face during the epidemic and promoting constructive ways of discussing the epidemic and possible stressors with children need to be considered.

Furthermore, our study showed that the quality of parent–child relationships, children’s emotional or behavioral problems, and time spent outside during the epidemic differed by parents’ educational status. In both cities, the proportion of children who had emotional problems, going outside, or whose parents expressed negative sentiments or discussed the epidemic in their presence or had more irritable attitudes and worse reported closeness during the epidemic were significantly higher in the groups with lower-educational levels than higher-education levels. These findings further highlight the need to decrease parents’ negative feelings or discussion in the presence of children and promote patience and increase closeness with children to benefit children’s mental health and lifestyle, particularly among parents with lower education levels. In previous studies, socially disadvantaged parents (in terms of education, employment, or economic status) were observed to have lower health literacy and awareness about their own or their child’s health risks, and may not be able to recognize negative changes in their child’s emotional health and lifestyles and provide timely support to their children^35,36^. In addition, socially disadvantaged parents may also be more likely to suffer psychological stresses and other health problems themselves, especially during the epidemic^37,38^. For example, a previous study observed that parents who had not returned to work due to the epidemic were more likely to experience higher stress and anxiety regarding job continuity and financial security^28^. In turn, highly stressed parents may be more irritable towards their children and more likely to express negative emotions in their child’s presence, which may lead to more frequent emotional and lifestyle problems among their children. Therefore, it is vital to deliver interventions targeting parents with lower educational attainment, providing them appropriate health education and psychological services to decrease the disproportionate emotional and behavioral problems their children face during the COVID-19 epidemic.

Finally, although we found significant differences in each indicator of parent–child relationships, externalizing and internalizing symptoms, and lifestyle behaviors in the two cities, the paucity of previous studies comparing regional differences in these factors makes it difficult to compare our estimates to current literature. However, a study by Duan et al. found that residence in Hubei province (compared with living in other provinces) was significantly associated with increased levels of depression among children aged 7–18 years^9^. Also, primary school students in Wuhan were more likely to have depressive symptoms than in Huangshi city^7^. Combined with our study, these findings might reflect differences in the severity of the epidemic or different stresses faced by residents in Wuhan and other cities, which influence the residents’ mental health.

Our study had a few limitations. First, there may be a degree of recall bias as we aimed to investigate changes in children’s lifestyle outcomes during the COVID-19 epidemic compared to the previous winter break (based on parent recall). Participants’ current mood states might affect recalling information during the last winter break. Second, most respondents were parents instead of the children themselves, which may lead to reporting bias. Third, our results may be influenced by a potential selection bias from the use of an online survey methodology. We sent multiple invitations to respondents who did not initially respond to our survey to reduce this bias. Finally, this was a cross-sectional study, and only associations can be inferred rather than causal relationships. There is a need for follow-up studies on the emotional and lifestyle changes of children and adolescents during public health emergencies such as the COVID-19 epidemic to validate our findings.

## Conclusion

Our study found that children had higher screen time, less physical activity, and worse sleep during the epidemic than in regular times. Around 10% of children were at risk of clinically significant emotional problems, and about half of the children had externalizing symptoms during the epidemic. Qualities like parental patient attitudes or closeness with their children and not the frequency of parent–child communication is more important for children’s mental or behavioral health. In addition, inequities in the prevalence of the previously mentioned study variables were found by parental education attainment. These findings imply that parents should be provided health education to improve their scientific knowledge about the epidemic, their ability to control adverse emotions (e.g., irritation), and to promote greater parent–child closeness. It is also important to improve the quality of communication between parents and children during and after the epidemic, especially for parents with lower educational levels.

## Supplementary Information

Supplementary Information.

## Data Availability

The datasets used and/or analyzed during the current study are available from the corresponding author on reasonable request.
