# Supplementary material for: Associations between parent–child relationship, and children’s externalizing and internalizing symptoms, and lifestyle behaviors in China during the COVID-19 epidemic
Source: Sci Rep. 2021 Dec 3;11:23375. doi: 10.1038/s41598-021-02672-7 (PMC8642441; doi:10.1038/s41598-021-02672-7)
Supplement: Supplementary file 1 — Supplementary Information. [file 41598_2021_2672_MOESM1_ESM.docx]

**Appendix A**

**Questionnaire: Online Survey of parent-child relationship, and children’s externalizing and internalizing symptoms, and lifestyle behaviors in China during COVID-19 epidemic**

1. **Socio-demographic characteristics**
2. Where do you live currently?

①Wuhan ②Shanghai ③None of the above (End the survey)

1. The gender of child/adolescent?

①Male ②Female

1. The age of child/adolescent? (years)

①3-5 ②6-9 ③10-14 ④15-17 ⑤None of the above (End the survey)

1. Has child/adolescent experienced fever or cough symptoms during the epidemic?

①Yes ②No

1. How many persons live together in your family (more than 4 days a week)?
2. What is your education level?

①Primary School or Below ② Junior high school ③Senior high school ④Three years college ⑤ Bachelor ⑥ Postgraduate or above

1. How do you perceive your family economic status?

① low ② low-middle ③ middle ④ high-middle ⑤ high

1. Have you returned to work after the Spring Festival?

①Yes, work at office ②Yes, work at home ③No ④Unemployed (including housewives, etc.)

1. Are there any confirmed or suspected COVID-19 cases in your neighborhood?

①Yes ②No ③Not clear

1. What’s your relationship with child/adolescent?
2. Mother ②Father ③Other (End the survey)
3. Who answered the questions about child/adolescent’s emotion and lifestyle changes?
4. Parent ②Child/adolescent
5. **Parent-child relationship during the COVID-19 epidemic**
6. During the COVID-19 epidemic, has child/adolescent actively asked about the epidemic?
7. Always ②Usually ③Sometimes ④Rare ⑤Never
8. During the COVID-19 epidemic, have you explained the epidemic to child/adolescent?
9. Always ②Usually ③Sometimes ④Rare ⑤Never
10. During the COVID-19 epidemic, have you expressed negative emotion or discuss the epidemic in front of child/adolescent?
11. Always ②Usually ③Sometimes ④Rare ⑤Never
12. During the COVID-19 epidemic, have you changed your attitude towards child/adolescent than usual?

①Become much more irritable ②Become a little more irritable ③Unchanged

④Become a little more patient ⑤Become much more patient

1. During the COVID-19 epidemic, has your closeness with child/adolescent changed than usual?

①Become much worse ②Become a little worse ③Unchanged ④Become a little better⑤Become much better

1. **Internalizing and externalizing symptoms of child/adolescent during the COVID-19 epidemic**
2. During the COVID-19 epidemic (before the start of school), has child/adolescent had any of the following emotional symptoms?

| No. | Emotional symptoms | Not True | Somewhat True | Certainly True |
| --- | --- | --- | --- | --- |
| 1 | Often complains of headaches, stomach-aches or sickness | 0 | 1 | 2 |
| 2 | Many worries or often seems worried | 0 | 1 | 2 |
| 3 | Often unhappy, depressed or tearful | 0 | 1 | 2 |
| 4 | Nervous or clingy in new situations, easily loses confidence | 0 | 1 | 2 |
| 5 | Many fears, easily scared | 0 | 1 | 2 |

1. During the COVID-19 epidemic, has child/adolescent had any of the following externalizing symptoms?

| No. | Externalizing symptoms | Always | Usually | Sometimes | Rare | Never |
| --- | --- | --- | --- | --- | --- | --- |
| 1 | Aggressive or stubborn behavior |  |  |  |  |  |
| 2 | Tantrums or meltdowns |  |  |  |  |  |

1. **Lifestyle changes of child/adolescent during the COVID-19 epidemic compared with usual**

**Please describe lifestyle status of child/adolescent during the COVID-19 epidemic:**

1. During the COVID-19 epidemic (before the start of school), how often did child/adolescent go outside?

①Never ②Less than once a week ③1-2 times a week ④3-5 times a week ⑤Nearly everyday

1. During the COVID-19 epidemic (before the start of school), how long was the screen time of child/adolescent per day?

① None ②less than one hour ③1-2 hours ④2-4 hours ⑤4 hours or more

1. During the COVID-19 epidemic (before the start of school), how many days did child/adolescent have moderate to vigorous exercise per week?

①0 ② 1 day ③ 2 days ④ 3 days or more

1. During the COVID-19 epidemic (before the start of school), how long did child/adolescent do moderate to vigorous exercise per day?

①Less than 30 minutes ②0.5-1 hour ③ 1 hour or more

1. During the COVID-19 epidemic (before the start of school), how many hours did child/adolescent sleep every day?

①9-11 hours ②8-9 hours ③7-8 hours ④5-7 hours ⑤Less than 5 hours

1. During the COVID-19 epidemic (before the start of school), did child/adolescent have difficulty getting to sleep at night?

①Never②1-2 times a month or less③1-2 times a week ④3-5 times a week ⑤Nearly everyday

1. During the COVID-19 epidemic (before the start of school), did child/adolescent wake up more than twice at night?

①Never②1-2 times a month or less③1-2 times a week ④3-5 times a week ⑤Nearly everyday

**Please describe lifestyle status of child/adolescent during the last winter break (before the epidemic):**

1. During the last winter break, how long was the screen time of child/adolescent per day?

① None ②less than one hour ③1-2 hours ④2-4 hours ⑤4 hours or more

1. During the last winter break, how many days did child/adolescent have moderate to vigorous exercise per week?

①0 ② 1 day ③ 2 days ④ 3 days or more

1. During the last winter break, how long did child/adolescent do moderate to vigorous exercise per day?

①Less than 30 minutes ②0.5-1 hour ③ 1 hour or more

1. During the last winter break, how many hours did child/adolescent sleep every day?

①9-11 hours ②8-9 hours ③7-8 hours ④5-7 hours ⑤Less than 5 hours

1. During the last winter break, did child/adolescent have difficulty getting to sleep at night?

①Never②1-2 times a month or less③1-2 times a week ④3-5 times a week ⑤Nearly everyday

1. During the last winter break, did child/adolescent wake up more than twice at night?

①Never②1-2 times a month or less③1-2 times a week ④3-5 times a week ⑤Nearly everyday

**Appendix B**

**Table B1 Equity (ratio between comparison groups) in parent-child relationship, children’s internalizing and externalizing symptoms, and lifestyle changes during the COVID-19 epidemic**

|  | **Ratio between low- and high-income groups**^*^ | | | | **Ratio between unemployed and office-working groups**^*^ | | | |
| --- | --- | --- | --- | --- | --- | --- | --- | --- |
|  | **Wuhan** | **P value** | **Shanghai** | **P value** | **Wuhan** | **P value** | **Shanghai** | **P value** |
| ***Panel A: Parent-child relationship indicators*** | | | | | | | | |
| Children actively asked about the epidemic^**^ | 1.12 | 0.148 | 0.93 | 0.124 | 1.45 | <0.001 | 1.18 | 0.001 |
| Parent explained the epidemic to children^**^ | 1.27 | <0.001 | 1.09 | 0.021 | 1.25 | <0.001 | 1.03 | 0.389 |
| Parent expressed negative sentiments or discussed the epidemic in their child’s presence^**^ | 1.50 | 0.004 | 1.08 | 0.457 | 1.55 | <0.001 | 1.13 | 0.201 |
| More irritable attitude towards children | 2.23 | 0.001 | 2.11 | 0.008 | 1.34 | 0.118 | 0.91 | 0.708 |
| Worse parent-child closeness | 2.97 | <0.001 | 0.78 | 0.462 | 2.74 | <0.001 | 0.81 | 0.567 |
| ***Panel B: Children’s internalizing & externalizing symptoms*** | | | | | | | | |
| Having emotional problems^#^ | 1.99 | 0.047 | 1.13 | 0.666 | 4.31 | <0.001 | 1.26 | 0.434 |
| Having externalizing symptoms | 1.58 | 0.001 | 0.94 | 0.473 | 1.62 | <0.001 | 1.29 | 0.002 |
| ***Panel C: Children’s lifestyle changes during the epidemic compared with normally*** | | | | | | | | |
| Going outside during the epidemic | 1.03 | 0.841 | 1.21 | 0.076 | 1.12 | 0.335 | 0.57 | <0.001 |
| More screen time per day | 1.44 | 0.036 | 0.94 | 0.639 | 2.16 | <0.001 | 1.03 | 0.817 |
| Less exercise per week or per day | 1.46 | 0.012 | 0.95 | 0.703 | 1.50 | <0.001 | 1.46 | 0.001 |
| Sleep less at night | 1.45 | 0.109 | 0.67 | 0.062 | 1.13 | 0.493 | 1.63 | 0.008 |
| More often had difficulty getting sleep or woke up > twice per night | 1.12 | 0.536 | 0.62 | 0.005 | 1.53 | 0.001 | 1.31 | 0.078 |

Note. ^*^ Ratios generated by dividing the proportion of the low-income group by the high-income group, or the unemployed group by the office-working group. The ratios indicate the gap between the comparison group - where 1 implies no gap, and P value are from Chi square or fisher’s exact tests (when applicable).

^**^ This is “Yes” category which includes response options of “always,” “usually,” and “sometimes” for three questions on the frequencies of parent-child communications about the COVID-19 epidemic.

^#^ One’s SDQ-Emotional Symptoms Score ≥5 for parent version or ≥7 for child version was considered to have substantial risk of clinically significant emotional problems (in short, having emotional problems).
